# Salivary Composition Is Associated with Liking and Usual Nutrient Intake

**DOI:** 10.1371/journal.pone.0137473

**Published:** 2015-09-04

**Authors:** Caroline Méjean, Martine Morzel, Eric Neyraud, Sylvie Issanchou, Christophe Martin, Sophie Bozonnet, Christine Urbano, Pascal Schlich, Serge Hercberg, Sandrine Péneau, Gilles Feron

**Affiliations:** 1 Université Paris 13, Equipe de Recherche en Epidémiologie Nutritionnelle (EREN), Centre d’Epidémiologie et Statistiques Paris Nord, Inserm (U1153), Inra (U1125), Cnam, COMUE Sorbonne Paris Cité, Université Paris 5, Université Paris 7, F-93017, Bobigny, France; 2 CNRS, UMR6265 Centre des Sciences du Goût et de l’Alimentation, F-21000 Dijon, France; 3 INRA, UMR1324 Centre des Sciences du Goût et de l’Alimentation, F-21000 Dijon, France; 4 Université de Bourgogne, UMR Centre des Sciences du Goût et de l’Alimentation, F-21000 Dijon, France; 5 Université de Toulouse; INSA,UPS, INP; LISBP, 135 Avenue de Rangueil, F-31077 Toulouse, France; 6 INRA, UMR792, Ingénierie des Systèmes Biologiques et des Procédés, F-31400 Toulouse, France; 7 CNRS, UMR5504, F-31400 Toulouse, France; 8 Department of Public Health, Hôpital Avicenne, F-93300 Bobigny, France; Tufts University, UNITED STATES

## Abstract

Salivary flow and composition have an impact on flavor perception. However, very few studies have explored the relationship between saliva, individual liking and usual dietary intake. The aim of our study was to evaluate the association of salivary flow and composition with both a liking for fat, saltiness and sweetness and the usual nutrient intake in an adult French population. Liking for fat, saltiness, and sweetness were inferred from liking scores obtained during hedonic tests on 32 food products among 282 French adults participating in the Nutrinet-Santé Study. Before assessing liking, resting saliva was collected. Standard biochemical analyses were performed to assess specific component concentrations and enzymatic activities. Dietary data were collected using three web-based 24h records. Relationships between salivary flow and composition, sensory liking and nutrient intake were assessed using linear regression. Total antioxidant capacity was positively associated with simple carbohydrate intake (β = 31.3, 95% CI = 1.58; 60.99) and inversely related to complex carbohydrate consumption (β = -52.4, 95% CI = -87.51; -19.71). Amylolysis was positively associated with both total (β = 0.20, 95% CI = 0.01; 0.38) and simple carbohydrate intake (β = 0.21, 95% CI = 0.01; 0.39). Salivary flow was positively associated with liking for fat (β = 0.14, 95% CI = 0.03; 0.25). Proteolysis was positively associated with liking for saltiness and for fat (β = 0.31, 95% CI = 0.02; 0.59; β = 0.28, 95% CI = 0.01; 0.56, respectively). Amylolysis was inversely associated with liking for sweetness (β = -10.13, 95% CI = -19.51; -0.75). Carbonic anhydrase 6 was inversely associated with liking for saltiness (β = -46.77, 95% CI = -86.24; -7.30). Saliva does not substantially vary according to a usual diet, except for carbohydrate intake, whereas the specific association between salivary flow/composition and sensory liking suggests the influence of saliva characteristics in food acceptance.

## Introduction

In recent decades, processed foods with high sensory attractiveness have become easily available and frequently consumed. Fat, sugar and sodium are responsible for the sensory attributes of numerous foods and greatly contribute to eating pleasure [1]. This could lead to overconsumption of such components and may be critically involved in risk of chronic disease [2]. Liking for fat, sweet or salty sensations and intakes of high-fat, salted and sweetened foods differ between individuals [3–6]. Thus, it is of interest to identify individual characteristics associated with liking and intake.

Taste and flavor perception affects food preferences and eating habits [3]. Previous studies reported that saliva might be involved in interindividual variation in sensory sensitivity, in addition to genetic polymorphism in taste receptors [7–15]. Indeed, salivary flow and composition (e.g. mucins, proline-rich proteins, sodium, amylolytic, proteolytic and lipolytic activities) have an impact on “in-mouth” perception of flavor, such as fat, sweetness, saltiness, astringency, bitterness and retronasal aroma. However, very few studies have explored the influence of salivary flow and saliva composition on individual taste liking or acceptance [11;16–18]. Previous works showed that salivary flow was positively associated with liking for fat and sourness, and that protein composition might be related to bitterness acceptance by infants. To our knowledge, only one study examined the relationship between saliva and liking for salty and sweet tastes; it found no significant association [18].

Literature on the relationship between saliva characteristics and usual dietary intake is scarce and has generally focused on animals. The few available studies in humans highlighted dynamic interactions between saliva and diet, suggesting plasticity of the salivary profile according to diet [19–23]. Thus, the relationship of salivary characteristics with liking and dietary intake represents a scientific challenge for better understanding why individuals eat fatty, sweet and salted foods that may be unhealthy when consumed in excess.

The aim of this study was to evaluate the association of salivary flow and composition first with usual nutrient intake (based on the hypothesis that nutrient intake could shape salivary characteristics) and then with liking for fat, saltiness and sweetness (based on the hypothesis that saliva characteristics could modulate liking). This study was conducted in a French adult population.

## Subjects and Methods

### Study population

Subjects were participants in the NutriNet-Santé Study, a large web-based prospective observational cohort launched in France in May 2009, with a scheduled follow-up of 10 years. It was implemented in a general population targeting Internet-using adult volunteers aged 18 or older. The study was designed to investigate the relationship between nutrition and health as well as determinants of dietary behavior and nutritional status. The design, methods and rationale have been described elsewhere [24]. Briefly, in order to be included in the cohort, participants had to complete an initial set of questionnaires assessing dietary intake, physical activity, anthropometry, lifestyle, socioeconomic conditions and health status. As part of their follow-up, participants complete the same set of questionnaires every year. Moreover, each month, they are invited to fill out complementary questionnaires related to determinants of food intake, nutritional and health status. In October 2010, participants already included in the cohort and living in a radius of 20 km from six sensory laboratories throughout France (Agen, Caen, Dijon, Rennes, Strasbourg, Surgères) were invited to participate to sensory tests for measuring liking for salty, sweet and fat sensations. Saliva collection was also performed during the sessions. These data were collected from January to May 2011.

This study was conducted according to guidelines laid down in the Declaration of Helsinki and all procedures were approved by the Institutional Review Board of the French Institute for Health and Medical Research (Institutional Review Board Inserm n° 0000388FWA00005831), the Ile-de-France III Ethics Committee of Tarnier-Cochin Hospital (n° 2010-A00182–37) and the French Data Protection Authority (Commission Nationale Informatique et Libertés n° 1148039, n° 908450 and n° 909216). Electronic informed consent was obtained from all participants. This study is registered in EudraCT (n°2013–000929–31).

### Data collection

#### Assessment of nutrient intake and nutritional status

Dietary data were collected using web-based 24 h dietary records. At enrollment and after one year, participants were invited to provide three 24 h records, randomly assigned over a two-week period (1 weekend day and 2 week days). The dietary record relies on a meal-based approach, recording all foods and beverages (type and quantity) consumed on all eating occasions [25]. First, the participant fills in the names of all food items eaten. Next, he/she estimates portion size for each reported food and beverage according to standard measurements or using photographs available via the interactive interface, taken from a validated picture booklet [26]. The nutritional values for energy and nutrients were estimated using published nutrient database [27]. Body mass index (BMI) was assessed using self-reported height and weight at enrollment.

#### Assessment of sensory liking

***Food products*.** Based on numerous pre-tests, 32 food products were selected: 10 for salt, 10 for fat, and 12 for sweets (S1 Table). Food products were selected to represent usual foods consumed by the French population. They included products of different consistencies (liquid / semi-liquid / solid) and different consumption temperatures. Each product had to be “homemade” (not commercially prepared) and easily reproducible. Each laboratory prepared its own food products following the recipes and protocols developed in the supervising laboratory in Dijon. These protocols allowed standardizing the basic ingredients and cooking conditions. For each food product, five levels of fat, saltiness and sweetness were prepared. Taste ingredients for fat were sunflower oil, pork fat or cream, for saltiness, NaCl and for sweetness sucrose or artificial sweeteners (sodium cyclamate and saccharin).

The medium level (level 0, L0) of saltiness, sweetness or fattiness, initially based on the most frequently used content in similar commercial products or in common recipes, was adjusted to conform to the preferences of approximately 50% of subjects. From this L0 level were derived the four other levels, by decreasing (levels L-1 and L-2) or increasing (levels L+1 and L+2) the concentration of the ingredient. In pre-tests, we validated that, for each food product, distribution of liking scores plotted against levels of fat, salt or sugar approximated normal distribution.

***Hedonic evaluation***. Sensory testing took place during six sessions at weekly intervals for most participants. Participants were informed that the aim of the study was to assess their liking for various foods, but they were unaware that the food products varied in fat, saltiness and sweetness. Each session was conducted at lunch time; participants were informed that they would taste and eat different products representing a full lunch, and thus were required not to eat before coming to the laboratory. Products were blind-tasted under a red light to mask potential color differences. For each food product, participants were given 5 samples at the same time, with each sample corresponding to one level of the target ingredient, and thus to one level of perceived intensity of the target taste. Participants had to taste and swallow each sample according to a specified order based on Williams’ Latin square for balancing position and a first-order carry-over effect. Subjects were required to entirely consume each sample before rating their subsequent hedonic feeling on a 9-point scale, with anchors “I do not like this at all” on the left and “I like this very much” on the right. Each product was served at the temperature at which it is usually eaten. The total amount consumed by each participant was approximately 500 g and 2510 kJ, equivalent to a light meal.

#### Saliva sampling

At the beginning of sessions, prior to assessment of sensory liking, resting saliva was collected by instructing the subjects to spit out the saliva regularly into a pre-weighed screw-cap cup over a period of 5 minutes. Subjects were requested not to eat, drink or smoke for at least one hour before collection of saliva samples. Cups were weighed and salivary flow rates were expressed in mL/min, assuming that one g of saliva corresponds to one ml. Immediately after collection, saliva samples were stored at-20°C. Then, they were transported frozen to a single laboratory where they were thawed and centrifuged for 30 minutes at 15,000 g to remove bacteria and cellular debris. Supernatants were then stored at-80°C until biochemical analysis.

#### Biochemical analyses of saliva samples

For each subject, the three biological replicates (three saliva samples) were analysed and mean values were calculated. Except for Elisa and HPLC measurements, biochemical analyses were performed as follows using the high-throughput enzyme screening facility ICEO, part of the Integrated Screening Platform in Toulouse (PICT, IBiSA, Toulouse, France). Samples organized into microtiter plates were managed using the integrated robotic Genesis RSP-200 platform (TECAN, Männedorf, Switzerland) allowing liquid transfer and spectrophotometric reading.

#### Protein concentration

Protein concentration (Prot, expressed in mg/ml) was obtained by the standard Bradford protein assay Quick Start (Bio-Rad, Hercules, CA, USA) using bovine serum albumin (Sigma-Aldrich, St. Louis, MO, USA) as a standard for calibration.

#### Enzyme activities

All enzyme activities were expressed in international enzyme activity units (U) per mg of saliva protein. One unit is defined as the amount of enzyme that catalyzes conversion of one micromole of substrate per minute. Lipolytic (lipolysis), proteolytic (proteolysis) and amylolytic (amylolysis) activities were determined as detailed below.

***Lipolysis*.** The buffer contained 50 mmol/l Tris-HCl, pH 7.5, 4 mmol/l CaCl2, 2 mmol/l EDTA (ethylenediaminetetra-acetic acid), 0.2% (weight/volume percent) NaTDC (sodium taurodeoxycholate), 1 mmol/l PMSF (phenylmethylsulfonyl fluoride), 1 mmol/l DTT (dithiothreitol) and 0.02% (weight/volume percent) sodium azide. The substrate solution was prepared by vortexing 19 volumes of the above buffer for 10 secondes with 1 volume of an ethanolic solution of 4-methylumbelliferyl 7-oleate (Sigma-Aldrich, St. Louis, MO, USA) for a final concentration of 1 mmol/l. The reaction was carried out in a microplate. The reaction started by adding 37.5 μl of saliva to 150 μL of substrate solution and 1.5 μl ethanol. An inhibition reaction was also conducted for each sample by adding 1.5 μL of 125 μmol/l ethanol solution of THL (tetrahydrolipstatin) instead of ethanol. The intensity of fluorescence was followed continuously for 30 min at 37°C (excitation filter 355 nm, emission filter 460 nm) using a Varian Cary Eclipse fluorescence spectrophotometer (Varian Inc., Palo Alto, CA, USA). Lipolysis was calculated from the difference between the average activity of slopes obtained for each sample without and with the lipase inhibitor THL. Activity was then read against a standard curve of umbelliferone. At each set of measurements, control of linearity and proportionality of the reaction was also performed with commercial lipase (Aspergillus Niger lipase, Sigma-Aldrich, St. Louis, MO, USA).

***Proteolysis***. Proteolysis was determined using a Pierce Fluorescent Assay kit (Pierce Biotechnology, Rockford, IL, USA). The assay is based on measurement of a fluorescein fragment released from a fluorescein-labelled casein substrate during proteolytic digestion. Fluorescence was followed for 60 min at 37°C (excitation at 494 nm/emission at 518 nm).

***Amylolysis*.** Amylolytic activity was determined using the CPNG3 assay kit (Biolabo, Maizy, France). The kit is based on measurement of hydrolysis of 2-chloro-4-nitrophenyl maltotrioside (CNPG3) into chloronitrophenol (CNP), maltotriose and glucose. The rate of formation of CNP, directly proportional to alpha-amylase activity, is measured at 405 nm (SUNRISE Reader, TECAN, Männedorf, Switzerland) against an amylase standard.

#### Carbonic anhydrase 6 and cystatin SN

Carbonic anhydrase 6 (CA6) and cystatin SN were quantified using ELISA kits from USCN Life Science Inc. (Hubei, PRC) and Cusabio (Hubei, PRC), respectively.

#### Sodium quantification

Saliva samples were diluted to 1/20 in Milli-Q-water (Millipore, Bedford, MA, USA) and filtered through a membrane (pore size = 0.45 μm, CIL, Sainte-Foy-La-Grande, France). The amount of sodium in saliva was determined by HPLC ionic chromatography using a Dionex ICS2500 ion chromatographic system (Dionex, Voisins le Bretonneux, France) and expressed in mmol/l. Quantifications were performed using calibration curves carried out with sodium standard solutions ranging from 0.1 to 10 mmol/l in 22 mmol/l sulfuric acid (r^2^ = 0.999).

#### Total antioxidant capacity

Total antioxidant capacity was determined using an ORAC assay kit (Zen-Bio, Research Triangle Park, NC, USA). The assay measures loss of fluorescein fluorescence over time due to peroxyl-radical formation induced by breakdown of 2,2’-azobis-2-methyl-propanimidamide, dihydrochloride (AAPH). Trolox [6-hydroxy-2,5,7,8-tetramethylchroman-2-carboxylic acid], a water-soluble vitamin E analog, serves as a positive control. It inhibits fluorescein fluorescence decay in a dose-dependent manner. The intensity of fluorescence was measured (excitation filter 485 nm, emission filter 538 nm) with a microtiter plate fluorometer (Victor 3-V, Perkin Elmer, Waltham, MA, USA). Antioxidant capacity of saliva was expressed as the Trolox equivalent.

All salivary variables were expressed as unit per mg of proteins.

### Statistical analysis

The present analyses focused on participants included in the Nutrinet-Santé cohort study who had completed at least three 24 h dietary records and for whom data for saliva composition and hedonic data were available. For each participant, nutrient intakes were calculated from the 24 h records, weighted according to the day (week or weekend). Diet-underreporting subjects were identified by the method proposed by Black [28]. Briefly, the basal metabolic rate (BMR) was estimated by Schofield equations [29] according to sex, age, weight and height collected upon enrollment in the study. BMR was compared to energy intake taking into account the physical activity level. A physical activity level of 0.88 was used to identify extremely underreporting subjects, and a physical activity level of 1.55 was used to identify other underreporting participants [28]. In 24 h records, the participant declared whether reported consumption was fairly representative of his/her usual diet or strongly differed due to specific events (illness, a social event, etc.). These comments, such as acute disease and information collected at enrollment regarding a current restrictive diet or a recent loss of weight (≥ 5 kg), were examined so as to identify specific conditions that might explain low energy intake. Participants who provided such information were not considered underreporters, whereas other underreporting participants were excluded from the analysis. In addition, erroneous quantities due to data entry errors were identified using day- and food-specific established thresholds.

Underweight/normal weight, overweight and obesity were defined according to the WHO classification for BMI, as BMI < 25, 25 ≤ BMI < 30 kg/m² and BMI ≥ 30 kg/m², respectively [30]. In terms of liking for fat, sweet and salty sensations for each food product, the optimal level of saltiness, sweetness or fattiness (Lopt) was determined through quadratic regression fitting hedonic ratings of the 5 variants of the food product. For each sensation and each subject, a liking value was computed by averaging Lopt values for the corresponding food products, with each Lopt weighted by the correlation coefficient (R) between observed and predicted data. If the quadratic regression could not be fitted for a food product, the latter was not taken into account in computation of the liking score of the corresponding sensation. When this occurred for more than 50% of food product sensations for a given subject, the liking score for this sensation was considered as missing.

Based on reviews of the literature, specific associations between salivary flow/ composition, sensory liking and nutrient intake were examined using analysis of covariance. The relationship between salivary variables and socio-demographic and weight characteristics were also performed using analysis of covariance. Since, in previous works [19;23;31–33], dietary composition appeared to modify saliva content, we performed regression models for which each saliva composition variable was a dependent variable and each dietary factor was an explanatory variable. Each model was adjusted for energy intake and for the sensory analysis laboratory at which it was done (one of six) to assess the association between saliva and diet. According to the literature, specific associations were studied: between intake of total, complex and simple carbohydrates and alpha-amylase content [23], between intakes of lipids, monounsaturated fatty acids, polyunsaturated and saturated fatty acids and lipolysis [34] and between sodium intake and salivary sodium content [35]. We also explored potential associations of salivary flow and total antioxidant capacity (TAC) with each nutrient intake (total, complex and simple carbohydrates, proteins, lipids, monounsaturated fatty acids, polyunsaturated and saturated fatty acids, and sodium) [11;19;36;37]. Since salivary flow and composition have an effect on taste perception, such as fattiness, sweetness, saltiness and bitterness [7;8;11;38], and consequently could impact taste liking, we performed models for which each liking score (fat, sweet and salty sensations) was a dependent variable and each saliva composition variable was an explanatory variable. Each model was adjusted for sensory laboratories, age and sex.

Two-sided tests and a P < 0.05 were used for statistical significance. A more conservative P value of 0.01 was also used for estimating the robustness of the results. Data management and statistical analyses were performed using SAS (version 9.1; SAS Institute, Inc., Cary, NC, USA) for regression models.

## Results

A total of 282 adults participated in sensory testing. We excluded 54 persons who had not provided at least three 24 h dietary records or who were underreporters, and 12 with missing data for all saliva variables, thus leaving 216 participants with available saliva analysis. The number of subjects for whom data were available was: 216 participants for flow measurement, 215 subjects for protein and amylolysis, 212 for lipolysis, 210 for TAC and proteolysis, 205 for sodium, 188 for CA6 and 185 for cystatin SN. Characteristics of the sample are presented in Table 1.

**Table 1:** Characteristics of the participants.

	n	Mean ± SD^1^or %
**Nutrient intake**		
Total energy intake (kJ/d)	214	10540.7 ± 3723.8
Lipid intake (g/d)	214	106.6 ± 41.
Saturated fatty acids (g/d)	214	47.6 ± 20.8
Monounsaturated fatty acids (g/d)	214	38.0 ± 15.5
Polyunsaturated fatty acids (g/d)	214	14.3 ± 6.6
Carbohydrate intake (g/d)	214	275.8 ± 118.9
Complex carbohydrate intake (g/d)	214	128.4 ± 50.0
Simple carbohydrate intake (g/d)	214	147.3 ± 84.8
Protein intake (g/d)	214	94.4 ± 28.2
Sodium intake (mg/d)	214	3677.4 ± 1753.4
**Salivary composition**		
Resting flow (ml/min)	216	0.8 ± 0.5
Total antioxidant capacity (U/mg of protein)	210	16790.1 ± 9030.9
Proteins (mg/ml)	215	0.6 ± 0.3
Proteolysis (U/mg of protein)	210	0.8 ± 1.3
Amylolysis (U/mg of protein)	215	122.9 ± 59.5
Lipolysis (mU/mg of protein)	212	1.0 ± 0.8
Sodium (mmol/mg of protein)	205	11.6 ± 7.9
Carbonic anhydrase VI (ng/mg of protein)	188	205.5 ± 180.7
Cystatin SN (ng/mg of protein)	185	859.5 ± 878.5
		
**Liking scores**		
Liking for fat sensation	209	-0.3 ± 0.6
Liking for sweet sensation	214	-0.2 ± 0.8
Liking for salty sensation	209	-0.1 ± 0.6
		
**Sex**		
Women		63.4
Men		36.6
**Age (years)**	216	49.6 ± 13.5
**Educational level**	216	
Elementary school		23.7
Secondary school		22.8
College graduate		53.5
**Smoking status**	216	
Current smoker		12.5
Former smoker		31.0
Never-smoker		56.5
**Body mass index (kg/ m2) categories**	216	
Normal (BMI ^2^ < 25)		56.0
Overweight (25 ≤ BMI^2^ < 30)		28.7
Obese (BMI^2^ ≥ 30)		15.3

1 SD: standard deviation

^2^ BMI: body mass index

### Associations between salivary flow/composition and nutrient intake

No significant association was found between salivary flow and nutrient intake (data not shown). TAC was positively associated with simple carbohydrate intake (β = 31.3, 95% CI = 1.58; 60.99), whereas it was inversely related to complex carbohydrate consumption (β = -52.4, 95% CI = -87.51; -19.71). Lipolysis was not associated with intake of lipids, monounsaturated fatty acids or polyunsaturated and saturated fatty acids (respectively, P = 0.11, P = 0.19, P = 0.73 and P = 0.18). Amylolysis was positively associated with both total (β = 0.20, 95% CI = 0.01; 0.38) and simple carbohydrate intake (β = 0.21, 95% CI = 0.01; 0.39), but not with complex carbohydrate intake (P = 0.77). Sodium content was not associated with the usual sodium intake (P = 0.78). Results regarding association between TAC and simple carbohydrate intake and those between amylolysis and total and simple carbohydrate intake did not remain significant when the more conservative p value of 0.01 was used. Regarding weight status, no significant difference was observed in salivary composition variables according to BMI or BMI classes (data not shown).

### Association between sensory liking and salivary flow/composition

Salivary flow was positively associated with liking for fat. Proteolysis was positively associated with liking for both salt and fat sensations. Amylolysis was inversely associated with liking for sweetness, while CA6 was inversely associated with liking for the salty sensation (Table 2). Results regarding amylolysis and CA6 did not remain significant when the more conservative p value of 0.01 was used.

**Table 2:** Associations between liking for fat, salt and sweet sensations and salivary variables^1^.

Saliva characteristics	Liking for fat		Liking for salt		Liking for sweet	
	β (95% CI)	*P*	β (95% CI)	*P*	β (95% CI)	*P*
Resting flow (n = 216)	**0.14 (0.03;0.25)**	**0.01**	0.09 (-0.02; 0.20)	0.10	0.06 (-0.016; 0.14)	0.12
Total antioxidant capacity (n = 210)	-704.00 (-2669.00; 1260.91)	0.48	-37.60 (-1973.22; 1898.14)	0.97	86.20 (-1362.19; 1534.55)	0.91
Protein concentration (n = 215)	0.05 (-0.01;0.11)	0.08	0.03 (-0.02; 0.09)	0.23	0.03 (-0.01; 0.07)	0.19
Proteolysis (n = 210)	**0.28 (0.01; 0.56)**	**0.006**	**0.31 (0.02; 0.59)**	**0.01**	0.10 (-0.11; 0.31)	0.28
Amylosis (n = 215)	-12.60 (-25.34; 0.13)	0.05	-8.79 (-21.43; 3.85)	0.17	**-10.13 (-19.51; -0.75)**	**0.03**
Lipolysis (n = 212)	-0.06 (-0.23; 0.11)	0.51	-0.14 (-0.30; 0.03)	0.11	-0.08 (-0.13; 0.12)	0.89
Sodium (n = 205)	-0.20 (-1.89; 1.56)	0.85	-0.55 (-2.33; 1.22)	0.54	-0.14 (-1.45; 1.18)	0.83
Carbonic anhydrase 6 (n = 188)	-25.00 (-64.60; 14.51)	0.21	**-46.77 (-86.24; -7.30)**	**0.02**	-20.41(-49.84; 9.01)	0.17
Cystatin SN (n = 185)	-0.03 (-195.65; 211.16)	0.77	-0.07 (-205.71; 207.99)	0.42	-0.03 (-88.01; 213.85)	0.60

^1^ All models were adjusted for sensory analysis laboratory site, sex and age.

## Discussion

The present findings highlight significant relationships between certain salivary composition variables (total antioxidant capacity and amylolysis) and the usual simple and complex carbohydrate intakes, thus emphasizing that salivary characteristics may vary to some extent according to the usual diet. Moreover, findings from this adult sample showed specific relationships between salivary flow/composition and liking for fat, salty and sweet sensations, suggesting the importance of saliva characteristics in food acceptance.

In this work, focusing first on the link between saliva and nutrient intake, we found a positive relationship between carbohydrate intake and salivary amylolysis. This relationship was significant with simple carbohydrates, but not with complex carbohydrates. Salivary amylase is involved in digestion of complex carbohydrates and previous works showed a positive relationship between human salivary amylase secretion and carbohydrate intake [39], and at a genetic level, that the number of copies of the alpha-amylase gene (AMY1) is higher in populations with high-complex carbohydrate diets than in populations with low-complex carbohydrate diets [23]. However, no work reported correlation between salivary amylase and simple carbohydrate consumption and therefore this result is unexpected.

No previous study in the literature had investigated the relationship between salivary TAC and usual dietary intake. One hypothesis for explaining the positive association between simple carbohydrate intake and total antioxidant capacity (TAC) observed in our study may be that subjects who ate more simple carbohydrates such as fructose had a significant rise in uric acid in plasma compared to those who ate only cornstarch as a source of carbohydrates [40]. More recently, a study conducted on overweight and obese subjects showed a significant increase in serum uric acid concentrations after consumption of fructose-rich beverages and a slighter but similar effect with glucose-rich drinks [41]. A similar population fed a low carbohydrate diet showed a decrease in uric acid in the blood after 24 weeks [42]. Since uric acid is the major contributor to salivary TAC [43–45], and since a high level of uric acid in plasma leads to high saliva TAC values [43], the higher saliva TAC observed in subjects with high simple carbohydrate intake compared to those with high consumption of complex carbohydrates might be explained by a higher level of uric acid in blood circulation, and consequently, in saliva fluid. Another explanation may be that carbohydrate intake, in particular simple carbohydrates, contributes to dental caries formation via their role in saliva pH acidification [46], and to development of dental plaque [47]. Dental plaque is related to development of bacteria, in particular anaerobic, [48;49] mainly controlled by the antioxidant properties of saliva [43]. Thus, differences observed in saliva TAC status in subjects with high intake of complex carbohydrates compared to simple carbohydrates might be due to the oral microflora composition, i.e. the higher the consumption of simple carbohydrates, the higher the TAC for controlling development of dental plaque.

Looking at the relationship between saliva and sensory liking, it was found that carbonic anhydrase 6, a zinc metalloprotein that has been described as a trophic factor for taste bud development [50], was inversely associated with liking for saltiness. Previous work had shown that patients with impaired taste acuity for NaCl, sucrose, HCl and urea had lower CA6 levels compared to healthy subjects [51]. Subjects with lower CA6 content may be less sensitive to salt and consequently might need a higher salt concentration to reach optimal liking (which is translated in our study by a greater liking for this taste). Interestingly, a recent study reported that perception of saltiness was associated with polymorphism of the *CA6* gene [52] suggesting a specific role of this protein in salt taste perception. However, to our knowledge, previous available studies did not evidence relationships between salt sensitivity and liking for saltiness that would support our findings [53–56].

The positive relationship between salivary flow and liking for fat was already reported in a previous study [11] in which evaluation of liking for fat was performed on a specifically designed emulsion. In that work, it was suggested that higher liking was associated with lower perceived intensity of fat due to higher salivary flow. Oral perception of fatty acids was considered unpleasant, and subjects with higher flow were better able to evacuate fat from the mouth, thereby decreasing unpleasantness. For milk and cream, other studies described a positive relationship between stimulated salivary flow and perception of fat level in the emulsion, but not with creaminess perception, a positive contributor to the liking for fat sensation [57;58]. The present study supports the hypothesis that salivary flow and fat liking in general may be linked, possibly through modulation of fat perception, while no specific relationship between salivary flow and liking for sweetness or saltiness was found.

The inverse relationship found between amylolysis and liking for sweetness could be related to the state of hunger. Indeed it was found that salivary alpha-amylase activity was negatively correlated with hunger [59]. In our study, saliva samples were collected at the beginning of the session, prior to rating of liking and state of hunger was not measured. Nevertheless, we could hypothesize that subjects who are more hungry (i.e. with lower amylolysis in our case) may be more prone to have optimum level of liking for higher levels of sweetness [60]. In addition, hydrolysis and thinning of starch viscous solutions proportionally to salivary amylase levels have been reported [61]. The modulation of sweetness perception, by amylolysis, during consumption of starchy matrices and consequently liking remains, however, uncertain.

The positive association between proteolysis and liking for saltiness and fat cannot be easily interpreted. In-the-mouth proteolysis and its control by endogenous protease inhibitors have been suggested to play a role in sensitivity to the bitter taste of caffeine [7]. Those authors suggested that proteolysis controls formation and structure of the mucosal pellicle, which covers the surface of the oral cavity, including the tongue and papillae. In the case of bitterness, higher proteolysis would result in a thinner or looser pellicle which would facilitate accessibility of taste molecules to the taste buds. Since this hypothesis is rather generic, it may be extrapolated to other tastes, and proteolysis could also modulate sensitivity to saltiness, for example. However, the fact that higher proteolysis would favor detection of salt with a positive impact on liking is somewhat contradictory to our previous findings in which carbonic anhydrase 6 would favor salt detection, but would have a negative impact on intake.

One of the strengths of our work lies in considering nutrient intake, liking and saliva all together in a diversified population using validated methods. However, interpretation of the present results must take into account several limitations. Participants were volunteers in a nutritional cohort study; they were in better health and had a healthier lifestyle than the general population [62], with lower intake of energy, fats, sodium and simple carbohydrates. Caution is therefore needed when interpreting and generalizing the results. Another limitation was that dietary intake collected by repeated self-administered web-based 24 h records might not be as accurate as data gathered via interviews by trained dietitians. However, a pilot study comparing our web-based 24 h record tool with the reference method (dietitian’s interview) showed strong agreement between the two methods, while suggesting that the web-based tool may even reduce judgment bias that would lead to food omission or underestimation of portion sizes for sweet/fatty items [25]. Moreover, one strength of the present study was the reliance on at least three dietary records provided in the course of one year, which enabled a reliable estimation of the usual diet [63]. The choice to measure unstimulated saliva and to contrast with liking for foods, when liking would be made when salivary flow is stimulated by the associated food cues could be a limitation. Indeed, stimulated saliva is of great importance in explaining bolus structure, or in-mouth aroma release as we recently showed [64]. However, we have shown on two different occasions that fat perception (intensity ratings, detection thresholds) and fat liking were most closely related to the characteristics of unstimulated saliva than that of stimulated saliva [11;37]. Furthermore, the type of stimulation has an impact on the nature of the resulting saliva. For example, salivary flow and composition is affected differently by different tastes [65] but even for the same basic taste (bitterness) by the molecule eliciting the taste [66]. Thus, in theory, saliva is unique to the product consumed. The experimental choice consisting of working on standardized (centrifuged heer) rather than crude saliva is also a deviation from the situation of food consumption. However, the question of saliva samples standardization has been discussed largely in a review from Schipper et al [67]. Differences between standardized and crude samples were indeed mentioned, but the authors also pointed out also the difficulty to work on crude saliva because of a rapid evolution of the sample after collection. As mentioned in the review, standardization can be done by a step of centrifugation to remove bacteria and cellular debris and storage at-80°C to arrest metabolism until subjected to biochemical analyses. In a context of the present work (multicentric study with saliva collection at different places), working on crude saliva immediately after collection was not feasible and standardization procedure was obligatory.

In conclusion, saliva does not substantially vary according to a usual diet, except for carbohydrate intake, whereas specific associations between salivary flow/composition and sensory liking suggest the influence of saliva characteristics in food acceptance. Although liking is linked to certain saliva characteristics and is an important determinant of food choices [3], we did not model taste perception or other factors, such as psychological factors, that contribute to dietary behavior and could play a role in the relationship between liking, saliva and dietary intake [1;3]. Further work exploring the interactions between these factors is needed to assess whether they modulate the relationship between physiological characteristics, hedonic responses and dietary patterns.

## Supporting Information

S1 Table Food products tested in each sensation. (DOCX)
