# Supplementary material for: Salivary Composition Is Associated with Liking and Usual Nutrient Intake
Source: PLoS One. 2015 Sep 4;10(9):e0137473. doi: 10.1371/journal.pone.0137473 (PMC4560437; doi:10.1371/journal.pone.0137473)
Supplement: S1 Table — (DOCX) [file pone.0137473.s001.docx]

**S1 Table.** Food products tested in each sensation.

| Sensation and  food product | Liqui, semi liquid solid |
| --- | --- |
|  |  |
| *Fat* |  |
| Leeks | solid |
| Tuna + mayo | solid |
| Mixed vegetables | solid |
| Polenta | semi liquid |
| Almond cake | solid |
| Cake | solid |
| Lentil puree | solid |
| Mashed potatoes | semi liquid |
| Soft white cheese | semi liquid |
| Zucchini + white sauce | solid |
| *Salt* |  |
| Green beans | solid |
| Carrot puree | semi liquid |
| Broccoli terrine | semi liquid |
| Mashed potatoes | semi liquid |
| Zucchini + white sauce | solid |
| Pasta + Bolognese | solid |
| Spinach cake | solid |
| Polenta | semi liquid |
| Vegetable soup | liquid |
| Salmon terrine | solid |
| *Sweet* |  |
| Chocolate milk | liquid |
| Soft white cheese | semi liquid |
| Corn flakes + milk | solid |
| Milk shake | liquid |
| Tea | liquid |
| Whipped cream | semi liquid |
| Chocolate custard | semi liquid |
| Verbena infusion | liquid |
| Strawberry syrup | liquid |
| Apple sauce | Semi liquid |
| Cake | solid |
| Orange juice | liquid |
